# Examining the Effects of Whole Crop Wheat Silage on Ewe Performance during Late Gestation Compared to Traditional Grass Silage across Three Prolific Breed Types

**DOI:** 10.3390/ani10091554

**Published:** 2020-09-02

**Authors:** Jonathan T. Higgins, Dermot Campion, Joe Jones, Stephen Lott, M. Bridget Lynch, Mary McEvoy, Fiona McGovern, Tommy M. Boland

**Affiliations:** 1 School of Agriculture and Food Science, University College Dublin, 4 D04 V1W8 Dublin, Ireland; joeboydjones95@gmail.com (J.J.); bridget.lynch@ucd.ie (M.B.L.); tommy.boland@ucd.ie (T.M.B.); 2 Germinal Ireland Ltd., Horse and Jockey, Co., E25 D286 Tipperary, Ireland; dermot.campion@germinal.com (D.C.); mary.mcevoy@germinal.com (M.M.); 3 School of Agriculture and Food Science, University College Dublin Lyons Farm, Lyons Estate, Celbridge, Naas, Co., W23 ENY2 Kildare, Ireland; stephen.lott@ucd.ie; 4 Animal and Grassland Research and Innovation Centre, Teagasc, Mellows Campus, Athenry, Co., H65 R718 Galway, Ireland; Fiona.McGovern@teagasc.ie

**Keywords:** late gestation nutrition, whole crop wheat silage, water intake, ewe performance, colostrum yield

## Abstract

**Simple Summary:**

Nutrition of the ewe during late pregnancy can impact the subsequent performance of the ewe and her lambs. In indoor lambing systems, grass silage is the predominant forage used but is of sub-optimal quality. This study examined the potential of whole crop cereal silage when offered to one of three prolific breed types in late pregnancy. With the exception of reduced crude protein intake on the whole crop wheat silage diets, nutrient intake or ewe body reserves were not affected by forage type. However, ewes offered whole crop wheat silage-based diets produced smaller lambs. Mule ewes produced lower volumes of colostrum in the first 18 h after lambing reflecting a lower nutrient intake during late pregnancy. Maternal nutrition and breed type merit careful consideration in prolific sheep production systems to ensure lamb welfare and survivability are not compromised.

**Abstract:**

Provision of adequate nutrient intake in late gestation of the ewe is an important determinant of dam and offspring performance. A 2 × 3 factorial design experiment examining two forage types, whole crop wheat silage (WCWS) or grass silage (GS) offered to one of three prolific breed types, (Belclare X, Lleyn X, Mule (Bluefaced Leicester × Blackface Mountain)), was conducted. Forage type had no impact on dry matter (DM) or metabolizable energy (ME) intake, body weight and body condition score change, or colostrum production (*p* > 0.05). Ewes offered WCWS had lower crude protein (CP) intake (*p* < 0.0001) and a lower combined litter weight (*p* < 0.05). Mule ewes consumed less DM, CP, (*p* < 0.05), and ME (*p* < 0.01) compared to Belclare X and Lleyn X ewes however, water intake per kg DM consumed did not differ with breed type (*p* > 0.05). Colostrum yield over the first 18 h postpartum was lower for Mule ewes compared to other breed types (*p* < 0.05). In conclusion, results from this study suggest nutrient concentration and balance as opposed to forage type is important for late gestation nutrition and breed type can impact feed intake and colostrum yield.

## 1. Introduction

Grazed grass is the most economical feed source for sheep in temperate regions of the world [1,2], however, climatic conditions limit grass growth during the winter months [3,4,5]. During this winter period, ewes in Ireland and elsewhere are often housed and offered conserved forage, predominantly grass silage [6,7,8], despite grass silage frequently being incapable of meeting the nutrient requirement of ewes, especially multiple bearing ewes, during late pregnancy [9,10]. Inadequate nutrient intake at this stage of the production cycle, which coincides with increased nutrient demand to support foetal development, can result in reduced colostrum production, milk production potential, and ewe body condition [11], and such periods of nutritional restriction may result in reduced lamb performance postpartum [12].

Whole crop wheat silage is an alternative feed source that has potential as a replacement for grass silage in the diet of the ewe during late gestation. Improvements in plant varieties [13], forage conservation [13], and availability [14] now make it a viable option. Studies have shown an increase in dry matter (DM) intake in beef [8,15] and dairy [16] cattle fed whole crop wheat silage compared to grass silage. Total feed cost € GJ·metabolisable energy (ME)^−1^ of whole crop wheat silage is lower than grass silage [2], with DM of whole crop wheat silage ranging from 250–450 g DM·kg^−1^ fresh weight (FW [17]) compared to grass silage, which averages 217 g DM·kg^−1^ FW [2]. This potentially provides the ewe with a more nutrient-dense feed source when rumen capacity is restricted in late gestation [18,19].

Maximising the number of lambs born per ewe per year is a key factor in profitable and sustainable sheep production [20], however the issue of dietary deficiency of nutrient supply is magnified with ewes of high prolificacy. With increased ewe prolificacy, the nutrient requirements, in particular the energy required by the ewe, also increases [21] during late pregnancy and lactation. Comparatively more expensive concentrate feed sources [2], with high energy and crude protein (CP) concentrations, are currently used to meet the ewe’s energy and CP requirements when forage alone cannot do so during the final trimester of gestation. There is a paucity of data in the literature examining the impact of feeding cereal silages to ewes during late pregnancy on subsequent ewe and lamb performance and its interaction with prolific breed type. It was hypothesised that (1) replacing grass silage (GS) with whole crop wheat silage (WCWS) in the ewe’s diet during late gestation would increase forage DM intake and thus reduce the requirement for concentrate feed, and (2) this response would be independent of prolific breed type. The objective was to examine the ewe and lamb performance to weaning when prolific breed types were offered WCWS or GS as the forage source during the final eight weeks of gestation.

## 2. Materials and Methods

All procedures performed in this experiment were approved by the Animal Research Ethics Committee at University College Dublin (UCD) and conducted under experimental license from the Health Products Regulatory Authority (HPRA) under the European directive 2010/63/EU and S.I. No. 543 of 2012. Each person who conducted procedures on experimental animals was authorised to do so by means of both individual authorisation and project authorisation from the HPRA. This experiment was conducted at UCD Lyons Research Farm, Celbridge, Naas, Co. Kildare, IE, W23 ENY2.

### 2.1. Pre-Experimental Ewe Management

Two hundred and thirty-four ewes, evenly divided between three breed types: Belclare X, Lleyn X, and Mule (Bluefaced Leicester × Blackface Mountain), were oestrus synchronized on 1 October 2018 using intravaginal progestogen pessaries (Chronogest and Folligon; Intervet Ireland, Ltd., Dublin, Ireland). Pessaries were inserted on day −14 (relative to ram introduction) and removed on day −2 prior to ram introduction, when each ewe received 500 i.u. of pregnant mare serum gonadotrophin (Chronogest and Folligon; Intervet Ireland, Ltd., Dublin, Ireland.) via intramuscular injection. Ewes were split into five separate mating groups, with each group balanced for breed type and joined with rams (Vendeen and Charollais) 36 h post pessary removal at a ratio of 1 ram:9 ewes. Post mating, all ewes grazed as a single group until 7 January 2019 (day 84 of gestation). Ninety ewes identified as twin bearing to the synchronised oestrus following ultrasound pregnancy diagnosis on day 74 post ram introduction were selected for the experiment and began a 7-day dietary acclimatisation on day 84 of gestation Prior to dietary acclimatisation all ewes were offered a grazed grass diet. Ewes allocated WCWS were gradually introduced to this feedstuff with 25% of their daily forage allocation comprising of whole crop wheat silage and 75% grass silage for the first two days of the acclimatisation period. The whole crop wheat silage allocation was increased by 25% and the grass silage allocation decreased by 25% every 2 days, until ewes were receiving 100% of the forage allocation of whole crop wheat silage by day 91 of gestation. Ewes allocated GS received 100% of their forage allocation as grass silage during this period.

### 2.2. Experimental Design

Forages (GS or WCWS) were offered to ewes of three prolific breed types (Belclare X, Lleyn X, and Mule) in a 2 × 3 factorial arrangement resulting in 15 animals per treatment. Before treatment allocation, ewes within breed type were blocked according to mating group and balanced for body weight (BW; 79.7 ± 7.78 kg), body condition score (BCS; 3.25 ± 0.42 units), and age at the start of the acclimatisation period. During the experimental period, commencing on day 91 of gestation, ewes were housed individually in pens measuring 1.1 m by 1.4 m, with 66 ewes housed on wooden slats and the remaining 24 ewes housed on expanded metal slats with floor type balanced across treatments.

### 2.3. Feeding Management Allowance

Ewes were individually offered 100% of their predicted requirement ME requirements, according to Agricultural and Food Research Committee (AFRC) [21] as amended by Robinson et al. [22], as follows:

Total energy requirement = maintenance + foetal requirements(1)

Maintenance energy requirement = (F + A)/Km(2)

Foetal energy requirement = Ec/Kc(3)

Energy content of the gravid foetus (Et) = log10(Et) = 3.322 − 4.979 − 0.00643t(4)

Daily energy retention in the foetus (Ec) = 0.25 Wo (Et (0.07372 − 0.00643t))(5)

in which: F = fasting metabolism, A = the activity allowance of the animal, Km = the efficiency factor for the utilization of ME for maintenance, Kc = the efficiency of energy utilization for conceptus gain, t = the number of days from conception, and Wo = the total expected litter weight at birth (kg).

The initial BW of the ewe, recorded at the beginning of the acclimatisation period, day 84 of gestation, and the total expected litter weight 11 kg [23] were constants in the equation to determine the ME requirement for each ewe during the course of the feeding period (day 91 to day 147 of gestation). Individual requirements were revised on a weekly basis to allow for the increasing foetal energy demands as gestation progressed.

Daily silage allowance was determined after the weighing of forage refusals at 07:00 h and 110% of the previous day’s intake was offered. When forage intake failed to meet ME requirements, ewes were individually offered concentrates to achieve 100% of their daily ME requirements. Forage was split fed at 08:00 h and 17:00 h. Concentrates were introduced on day 119, on an individual ewe basis when the ewe ME intake from forage was insufficient to meet 100% of ME requirements. Concentrates were offered daily at 09:00 h when daily concentrate allocation was less than 500 g but larger quantities were offered in two equal allocations at 09:00 h and 18:00 h. The offered concentrate had a CP concentration of 19.4% and contained 23% maize, 23% barley, 21% soya bean meal, 12.5% soya bean hulls, 10% distillers’ grains, 6% sugar cane molasses, 2.8% minerals, and 1.7% limestone flour, on a DM basis. Harvest period for both forages was July 2018 with the grass crop allowed a 24 h wilt. Forages were preserved under anaerobic conditions in two separate silos and no additive was used in their preservation. Representative samples of each forage were collected daily and concentrate samples were collected weekly and frozen at −20 °C. Forage samples were pooled by week, giving eight forage samples of each type and four concentrate samples for proximate analysis. The chemical composition of feedstuffs offered is outlined in Table 1.

Ewes had continuous access to clean, fresh drinking water with voluntary water intake recorded from day 113–115, day 126–128, and day 141–143 of gestation. Voluntary water intake was recorded over a 48 h period. For each ewe, the initial volume of water provided, all additional water added, and remaining water after 48 h was weighed, and then the total volume consumed per 48 h was calculated.

### 2.4. Ewe Measurements

Ewe BW was measured on day 84, 91, and 119 of gestation, 24 h postpartum, and on day 21, 42, and 98 of lactation using electronic weigh scales (Tru-Test Group, Auckland, New Zealand) and electronically recorded (Tru-Test Group, Auckland, New Zealand). Ewe BCS was assessed at each weighing date by a single trained operative according to a 5-point scale as described by Jefferies [24]. Ewe apparent efficiency of energy utilisation was estimated as:

(Ewe predicted required daily ME intake for actual CLW^Ŧ^/Ewe actual daily ME intake) × 100 (6)

^Ŧ^CLW = combined litter weight produced (7)

### 2.5. Lambing Data

Ewes lambed in their individual pens with 24 h supervision provided. Ewes and their lambs remained in individual pens for 48 h postpartum. After the first lamb was delivered, an udder cover was placed on the ewe to prevent suckling and to facilitate determination of colostrum yield at 1, 10, and 18 h postpartum. Within an hour of parturition, the umbilical cord of each lamb was sprayed with a 10% iodine solution to aid in the control of erysipelas polyarthritis (“joint-ill”).

Six ewes gave birth to triplets and were removed from the experiment, one in each of the Belclare X and Lleyn X WCWS treatments and two in each of the Belclare X GS and Mule WCWS treatments. One ewe in the Mule GS treatment gave birth to one mummified lamb, so was excluded from the study. One ewe in each of the Mule WCWS and Lleyn X GS groups subsequently could not rear their twin lambs so were removed from postpartum data collection.

All ewes were managed as a single group postpartum. Ewes and lambs remained group-housed for 9 days postpartum in straw bedded pens in groups of 15 ewes (plus their lambs) where they were offered GS ad libitum and 500 g of concentrates twice daily. At turnout, concentrate supplementation was discontinued and ewes were offered a grass-only diet consisting predominantly of perennial ryegrass.

### 2.6. Colostrum Sampling

All ewes were hand milked at 1, 10, and 18 h postpartum as described by Boland et al. [25]. Total yield was recorded after each milking and total yield to 18 h was calculated. At each time point, a 30 mL sample of colostrum was collected for determination of total solid (TS) and CP concentration. All samples were frozen at −20 °C until required for analysis. Each lamb was stomach tubed with its dam’s colostrum at a rate of 20 to 50 mL per kg lamb birth weight. Dependent on colostrum yield, each lamb received the maximal volume of colostrum available up to 50 mL per kg lamb birth weight. Where colostrum yield was insufficient to meet the 20 mL per kg lamb birth weight threshold, the lamb(s) received substitute pooled colostrum.

### 2.7. Lamb Parameters

Lamb birth weight and gender was recorded within 1 h of birth and lamb BW was subsequently recorded at 3, 6, and 14 weeks postpartum. With the exception of the measurement at 1 h, BW was measured using electronic scales (Prattley, Temuka, Canterbury, New Zealand) and electronically recorded (Tru-Test Group, Auckland, New Zealand). Lamb average daily gain (ADG) was calculated by regression of BW on time.

### 2.8. Chemical Analysis

Nutrient concentrations, unless stated, are displayed as g·kg^−1^ DM. Dry matter concentration (g·kg^−1^ FW) of feedstuffs was determined by drying the samples at 55 °C for 72 h in ventilated oven with forced air circulation. Dried samples were ground through a 1 mm sieve (Christy and Norris Hammer Mill, Chelmsford, England) before DM correction via 105 °C for 16 h in an incubator oven. Nitrogen concentration was determined using a LECO TruSpec N instrument (Leco Instruments, UK, Ltd., Stockport, UK) according to the method of Dumas (method number 990.03; Association of Official Analytical Chemists [26]). Nitrogen concentration of the samples was multiplied by 6.25 to determine CP concentration as per Kjeldahl [27]. All samples were analysed for neutral detergent fibre (NDF) and acid detergent fibre (ADF) according to the method of Van Soest et al. [28] using an ANKOM200 Fiber Analyzer (ANKOM TECHNOLOGY, Macedon, New York) with WCWS and concentrate samples being pre-treated with heat-stable amylase prewash. Acid detergent lignin was subsequently carried out once both NDF and ADF was complete by soaking the samples in 72% sulphuric acid for 3 h in a DAISY*^II^* Incubator (ANKOM TECHNOLOGY, Macedon, New York) and then triple rinsing with distilled water before drying samples at 100 °C overnight. Ash concentration was determined by complete combustion in a muffle furnace at 550 °C for 5 h. Organic matter digestibility (OMD) was estimated using the in-vitro method as per Tilley and Terry [29] via the DAISY*^II^* Incubator (ANKOM TECHNOLOGY, Macedon, New York). The gross energy concentration (MJ·kg^−1^ DM) of the samples was determined using bomb calorimetry (Parr 1281 bomb calorimeter; Parr Instrument Company, Moline, IL) according to the method outlined by Porter [30], and the ME was calculated using the following equation from [21]:

ME = (0.016 × Digestible organic matter digestibility)(8)

Ether extract concentration was determined using light petroleum ether and Soxtec instrumentation (Tecator, Hillerod, Sweden). Starch concentration of WCWS and the concentrates was determined using the Megazyme total starch assay procedure (method number 996.11; Association of Official Analytical Chemists [26]; Megazyme, Bray, Co. Wicklow, Ireland).

Colostrum was warmed to 38 °C in a water bath and a subsample was analysed for TS and CP concentration (g·L^−1^). Total solid concentration was determined using a direct forced-air method where 2.5 g of colostrum was placed in an oven for 4 h at 105 °C [31]. The nitrogen concentration was determined using a LECO TruSpec N instrument (Leco Instruments, UK, Ltd., Stockport, UK) according to the method of Dumas (method number 990.03; Association of Official Analytical Chemists [26]). The nitrogen concentration in the colostrum was multiplied by 6.38 to determine CP concentrations as per Kjeldahl [27].

### 2.9. Statistical Analysis

Data were analysed as a complete randomised block design using the mixed model and glimmix procedures (PROC MIXED and PROC GLM respectively) in SAS (SAS, version 9.4, Inst. Inc., Cary, NC, USA). Individual ewe was the experimental unit for all parameters. Data distributions were analysed to fit the assumptions of normality using the UNIVARIATE procedure. The fixed effects of treatment, time (as the repeated Constant; for repeated measures), forage type, breed type, the 2-way interactions of breed type × time, forage type × time, breed type × forage type, and the 3-way interaction of breed type × forage type × time were included in the model. Any variable with a *p*-value of >0.25 was removed. Ewe ME from forage, apparent efficiency of energy utilisation, water intake, BW, BCS, colostrum yield and composition at each time point, colostrum intake per lamb at each time point, and lamb BW were all treated as repeated measures. The repeated measures were fit using variance-covariance structures with the lowest Bayesian Information Criterion used to select the most appropriate variance-covariance structure. Ewe feed and nutrient intakes, BW and BCS percentage changes, total colostrum yield for the first 18 h postpartum, yield of colostrum per kg BW at 18 h postpartum, yield of colostrum per kg of combined litter weight (CLW), colostrum produced per hour, and lamb ADG were analysed using the glimmix procedure. All data presented in the tables are expressed as least squares means ± SEM. The probability value, which denotes statistical significance, is *p* ≤ 0.05, and tendencies are denoted by 0.05 < *p* ≤ 0.10.

## 3. Results

### 3.1. Feed and Nutrient Intake

The effect of forage type and breed type on nutrient intake and apparent efficiency of energy utilisation are presented in Table 2. Mean daily DM and ME intake were unaffected by forage type (*p* > 0.05), while ewes offered GS achieved higher mean daily intakes of CP and NDF (*p* < 0.001). Within breed type, Mule ewes achieved lower mean DM, CP, (*p* < 0.05) ME, and NDF (*p* < 0.01) intakes than Belclare X and Lleyn X ewes, which did not differ (*p* > 0.05). When expressed per kg ewe BW, DM and nutrient (ME, CP, NDF) intakes followed the same pattern as absolute DM and nutrient intakes (*p* < 0.05).

The mean percentage of ME intake achieved from forage was higher for ewes offered GS (*p* < 0.001), however the apparent efficiency of energy utilisation was lower for these ewes (*p* < 0.01). Belclare X and Lleyn X ewes achieved a higher percentage of ME intake from forage than Mule ewes (*p* < 0.05). The apparent efficiency of ME utilisation was higher (*p* < 0.001) for Mule ewes than Belclare X or Lleyn X, which did not differ (*p* > 0.05).

There was a forage type by breed type interaction for DM intake as Mule ewes offered WCWS consumed less DM than Belclare X (*p* < 0.0001) or Lleyn X (*p* < 0.05) ewes. No between-breed-type differences were detected for ewes offered GS. There was a forage type by breed type interaction for mean ME intake as Mule ewes offered WCWS consumed less ME than Belclare X ewes (*p* < 0.0001), Lleyn X ewes (*p* < 0.01) offered WCWS and Mule ewes offered GS (*p* < 0.05). Belclare X ewes offered GS consumed less ME than Belclare X ewes offered WCWS (*p* < 0.05).

### 3.2. Water Intake

The effect of forage type and breed type on water intake is presented in Table 3. Voluntary water intake was lower for ewes offered GS (1.8 L·day^−1^) than ewes offered WCWS (3.8 L·day^−1^; *p* < 0.0001). Ewes offered GS consumed more water as a component of their feedstuff than ewes offered WCWS (*p* < 0.0001), however total water consumption or water intake per kg DM intake did not differ with forage type (*p* > 0.05). Voluntary water intake of Mule ewes was lower than Lleyn X ewes (*p* < 0.05), with Belclare X ewes, intermediate (*p* > 0.05). Water consumed as a component of the feedstuff was lower (*p* < 0.05) for Mule ewes than Belclare X and Lleyn X ewes, which did not differ (*p* > 0.05). While total water intake was lower for Mule ewes than Lleyn X ewes (*p* < 0.01), with Belclare X ewes intermediate (*p* > 0.05), water intake per kg of DM intake did not differ between breed type (*p* > 0.05).

Voluntary water intake for ewes offered GS (*p* < 0.001) and WCWS (*p* < 0.01) increased as gestation progressed. Water consumption as a component of feedstuff did not differ between day 113–115 and day 126–128 of gestation (*p* > 0.05) for ewes offered GS, however water consumption as a component of feedstuff was lower on day 141–143 of gestation than both earlier time points (*p* < 0.001). Water intake as a component of feedstuff for ewes offered WCWS reduced as gestation progressed (*p* < 0.05).

Voluntary water intake by all breed types was lower between day 113–115 of gestation (*p* < 0.0001) than either day 126–128 or day 141–143 of gestation, which did not differ (*p* > 0.05). Belclare X and Lleyn X ewes’ water consumption as a component of feedstuff did not differ from day 113–115 to day 126–128 of gestation (*p* > 0.05) but was lower on day 141–143 of gestation (*p* < 0.0001). Mule ewes’ water consumption as a component of feedstuff was higher on day 113–115 of gestation than day 126–128 and day 141–143 of gestation (*p* < 0.05).

Total water consumption for ewes offered GS on day 113–115 of gestation was lower (*p* < 0.0001) than day 126–128 and day 141–143 of gestation, which did not differ (*p* > 0.05). Total water consumption for ewes offered WCWS was lower on day 113–115 of gestation compared to day 126–128 of gestation (*p* < 0.0001) but water intake on day 141–143 of gestation was lower than day 126–128 of gestation (*p* < 0.001) but not different from day 113–115 of gestation (*p* > 0.05). Total water consumption for all breed types for day 113–115 of gestation was greater than day 126–128 of gestation (*p* < 0.001). Water intake per kg of DM intake was not influenced by forage type or breed type.

### 3.3. Ewe BW and BCS

The impact of forage type and breed type on ewe BW and BCS is presented in Table 4. Individual timepoint measurements of BW and BCS and percentage change in BW and BCS from pre-experiment to both lambing and weaning did not differ with forage type or breed type (*p* > 0.05).

Body weight of ewes offered GS declined from pre-experiment to parturition (−2.41 kg; *p* < 0.01), however for ewes offered WCWS no significant decline was recorded (−2.07 kg; *p* > 0.05). Ewe BW reduced between parturition and weaning (*p* < 0.0001) for ewes offered either forage types. Pre-experimental BW and BW at parturition did not differ for any breed type but reductions in BW from parturition to weaning occurred for all breed types (*p* < 0.01).

Body condition score of ewes offered either GS or WCWS reduced from pre-experiment to parturition (−0.32 and −0.30 BCS units, respectively; *p* < 0.01) while no decline in BCS occurred from parturition to weaning. Within breed type, Belclare X and Mule ewes showed reductions in BCS from pre-experiment to parturition (−0.35 and −0.39 BCS units respectively; *p* < 0.05).

### 3.4. Combined Litter Weight

Ewes offered GS produced lambs with a higher CLW than ewes offered WCWS (*p* < 0.05) while CLW was not influenced by breed type (*p* > 0.05). There was an interaction between forage type and breed type for CLW (*p* < 0.05). Mule ewes offered WCWS produced lambs with a lower CLW than Mule ewes offered GS (*p* < 0.01), with no difference between silage type for Belclare X or Lleyn X ewes (*p* > 0.05). Within forage type Mule ewes offered GS produced lambs with a higher CLW than Belclare X ewes (*p* < 0.05) while within those offered WCWS, Belclare X ewes produced lambs with a higher CLW than Lleyn X ewes (*p* < 0.05). When comparing kg of CLW per kg of ewe BW at parturition (kg CLW/kg ewe BW) as a percentage, ewes offered GS had a higher percentage to ewes offered WCWS, 13.32% and 12.48%, respectively (*p* < 0.05; Table 4). No breed type or forage type × breed type differences were detected (*p* > 0.05).

### 3.5. Colostrum Yield and Composition

The effect of forage type and breed type on colostrum yield and composition are presented in Table 5. Forage type offered had no effect on colostrum yield at 1, 10, or 18 h or total colostrum yield to 18 h postpartum (*p* > 0.05). However, ewes offered GS had lower colostrum yield at 1 h compared to 10 h (*p* < 0.05) whereas colostrum yield of ewes offered WCWS did not differ between time points (*p* > 0.05). Colostrum yield at 1, 10, or 18 h postpartum was not affected by breed type (*p* > 0.05) but over the combined 18 h period, Belclare X ewes had higher (*p* < 0.05) colostrum yields than Mule ewes, with Lleyn X ewes intermediate (*p* > 0.05). No increases in colostrum yield at individual time points were observed for Belclare or Lleyn X ewes (*p* > 0.05) however, Mule ewes had increased colostrum yields at 10 and 18 h compared to 1 h postpartum (*p* < 0.01).

Colostrum production per kg ewe BW, per kg lamb birth weight, or colostrum production per hour from 1 to 10 h or 10 to 18 h postpartum did not differ with forage type (*p* > 0.05; Table 5). Mule ewes produced less colostrum per kg of lamb birth weight than other breed types (*p* < 0.05) but other colostrum production variables were not impacted by breed type (*p* > 0.05).

Ewes offered WCWS produced colostrum with higher TS concentration at 10 h postpartum than ewes offered GS (*p* < 0.05). Colostral CP concentration did not differ with forage type at any time point (*p* > 0.05). Mule ewes produced colostrum with higher TS at 10 h postpartum than Lleyn X ewes (*p* < 0.05) and higher CP concentration than Lleyn X ewes at 1 h (*p* < 0.05) and 10 h (*p* < 0.01) postpartum. Total solid and CP concentrations decreased as duration postpartum increased regardless of forage or breed type (*p* < 0.0001).

The effect of forage type and breed type on lamb colostrum intake is presented in Table 6. Forage type had no impact on colostrum intake per kg lamb BW at 1, 10, or 18 h postpartum (*p* > 0.05), with lambs born to ewes offered GS receiving a higher total colostrum intake compared to lambs born to ewes offered WCWS over the first 18 h postpartum (*p* < 0.05). Lambs born to Mule ewes received less colostrum per kg BW at 1 h postpartum than lambs born to Belclare X ewes (*p* < 0.001) and in total to 18 h postpartum than all other lambs (*p* < 0.01).

Interactions between forage type and breed type were observed for total colostrum intake per kg of lamb birth weight during the first 24 h of life (total colostrum·kg^−1^ lamb birth weight). Within forage type, lambs from Mule ewes offered GS received lower volumes of colostrum than lambs from Belclare X (*p* < 0.0001) and Lleyn X (*p* < 0.0001) ewes offered GS. Within breed types, lambs from Mule ewes offered GS received lower volumes of colostrum than lambs from Mule ewes offered WCWS (*p* < 0.05), lambs from Belclare X and Lleyn X ewes did not differ between forage type offered (*p* > 0.05).

### 3.6. Lamb BW and Growth Rate

The effects of forage type and breed type on BW of lamb at birth, 3, 6, and 14 weeks postpartum and lamb growth rate to weaning are presented in Table 7 and were unaffected by forage type, breed type, or the interaction between forage type and breed type (*p* > 0.05).

## 4. Discussion

Late gestation nutrition is vital to ensuring a productive sheep enterprise by maximising flock performance [11,23]. The hypothesis stated that (1) replacing GS with WCWS in the ewe’s diet during late gestation would increase forage DM intake and thus reduce the requirement for concentrate feed and (2) this response would be independent of prolific breed type. Forage type offered did not impact DM intake with a distinct breed type difference occurring, leading to the rejection of these hypotheses.

### 4.1. Feed and Nutrient Intake

All ewes in the current study were offered 100% of ME requirements, hence ME intake did not differ with forage type in contrast to previous studies that reported increased energy intake when WCWS was fed [8,15,16,32], as no intake restrictions were applied in those studies. Differences in nutrient intakes reported in the current study reflect differences in nutrient concentration (CP, NDF, and starch) between the two forages offered, however ewes on all treatments received a minimum of 100% of their CP requirements according to AFRC [21]. Declining intake capacity of ewes in the final weeks of gestation can be partly compensated for by increased digesta passage rate through the rumen [33] along with providing ewes with high concentrations of ME, CP, and starch in feedstuffs such as cereal grains [34] otherwise ewes will mobilise their body fat reserves [11]. The current study found that ME intake from forage alone declined more rapidly in ewes offered WCWS compared to GS, with ewes offered WCWS receiving 8%, 11%, 13%, and 5% less ME from forage in the final four weeks of gestation, respectively, compared to ewes offered GS, likely due to the lower OMD concentration in WCWS. Therefore, ewes offered GS achieved a higher percentage of total ME intake from forage and required less concentrate supplementation to meet energy requirements. This reflects the nutritional composition of the forages offered in the current study.

While studies in sheep are absent, the literature involving beef cattle is contradictory in relation to the impact of whole crop wheat silage on feed efficiency expressed as carcass gain per ME intake (g·MJ^−1^), with Walsh et al. [8] reporting improvements in feed efficiency but Mc Geough et al. [15] reporting no improvements in feed efficiency of beef cattle fed whole crop wheat silage compared to grass silage. The current study had a higher apparent efficiency of energy utilisation (calculated as ewe predicted required daily ME intake for actual CLW/ewe actual daily ME intake) when WCWS was offered in agreement with Walsh et al. [8] who attributed this increased animal efficiency to the high starch concentration of whole crop wheat silage, which is more digestible than NDF, the latter being present in lower concentrations in WCWS than in GS in the current study.

The lower DM (and subsequent nutrient) intake in Mule ewes compared to both Belclare X and Lleyn X ewes reported in the current study aligns with Arnold [35] who reported that Leicester X ewes had lower OMD intake per kg of BW than Merino and Dorset horn ewes. The dams of the Mule ewes in the current study were Scottish blackface, which Claffey et al. [36] reported had lower intakes (kg FW) compared to Scottish blackface × Texel lambs. Regarding the findings from the current study, Arnold [35] and Claffey et al. [36] suggest that Mule ewes are genetically predisposed to lower feed intakes compared to other breed types. However, the performance of the Mule ewes remained similar for both BW and BCS compared to the other breed types, which had increased feed intake. This suggests that Mule ewes potentially have reduced rumen passage rate of forage due to lower volumes consumed resulting in increased digestibility of the ingested forage [37] thereby increasing efficiency of energy utilisation in late pregnancy compared to the other breed types used in the current study.

### 4.2. Water Intake

Water has four main functions in the ewe; elimination of waste digestion products, regulation of blood osmotic pressure, main component of secretions (saliva and milk), and thermoregulation [38]. Ewe total water intake fluctuates depending on metabolic state, temperature, size of animal, wool depth, and DM intake Forbes [39] with further differences in total water intake during late gestation influenced by dietary composition [25] and litter size [39,40]. In the current study, the lower voluntary water intake of ewes offered GS is directly linked to the lower DM concentration of GS compared to WCWS, an observation in agreement with Walsh et al. [8], Mc Geough et al. [15], Burke et al. [16], and Gunal et al. [32] and concurrent increased dietary water intake. Total water intake per kg DM consumed remained similar between forage type during the final five weeks of gestation and as explained by Forbes [39] was a result of similar litter size, ewe BW, DM intake, and total water intake [39]. However, higher volumes of water were required in the current study compared to previous studies [39,40]. Wildeus et al. [41] reported differences in water intake between breed types. Similarly, the current study reported differences with Mule ewes consumed lower total water compared to Lleyn X ewes resulting from reduced DM intake with days in gestation, litter size, and ewe BW remaining similar, resulting in total water intake per kg DM consumed to remain unaltered. Forbes [39] indicated that during the final four weeks of gestation there is an increased requirement for water within the ewe increasing the total water per kg DM consumed.

### 4.3. Ewe BW and BCS

The lack of impact of forage type on ewe BW and BCS is in contrast to previous findings where moving to whole crop wheat silage diets elicited increases in animal BW and condition [8,15]. In the current study, treatments were isoenergic and ME allowance was limited to 100% of predicted requirements demonstrating that available energy and nutrients, rather than forage type, dictates ewe performance in late pregnancy [42].

The lack of variation in ewe BW at individual timepoints over the course of the study across breed type is somewhat surprising given the lower nutrient intake by Mule ewes. While daily differences in ME intake were relatively small (1.4 MJ ME per day), this accumulated to a reduction of ME intake over the feeding period of 78.4 MJ per ewe equivalent to 6 kg of concentrate DM. However, using BW as a sole indicator of the ewes’ body reserves is a poor assessment of ewe condition [23] and therefore BCS was also used in this assessment. McGovern et al. [11] reported higher BW and BCS at parturition for ewes with higher ME intake in late gestation though the magnitude of ME intake differences reported by McGovern et al. [11] was greater than that in the current study. The current findings show that even in situations of reduced energy intake, relative to Belclare X and Lleyn X, Mule ewes can maintain maternal reserves and lamb birth weight, two of the major late pregnancy energy sinks although negative impacts on colostrum production were recorded.

### 4.4. Combined Litter Weight

Previous studies reported that CLW is a poor indicator of adequate ewe nutrition [43,44] with contradictory reports of the impact of modest (±20%) variations in late pregnancy energy intake on subsequent lamb birth weight [11,23]. However, studies reporting 35% differences in energy intake have reported differing lamb birth weights [45]. The current study had no variation in energy intake between forage types, but ewes offered GS had higher CLW than ewes offered WCWS. This is attributed to the variation in CP concentrations between forage types with ewes offered GS having 20.8% higher CP intake per ewe than those offered WCWS. This was in agreement with Ocak et al. [46] who reported significantly higher birth weight of lambs in ewes on diets consisting of up to 40% higher CP concentrations during gestation.

Forage type by breed type interactions occurred as Mule ewes offered GS had heavier CLW than Mule ewes offered WCWS resulting from increased ME and CP intake in the present study, which is in agreement with [45,47] and [46] respectively. Previous research has reported breed type effects on lamb birth weight [48] contrary to the findings of the main breed type effect of the current study. Therefore, the higher CLW produced between treatments of Mule ewes offered GS compared to Belclare X ewes offered GS with similar DM and ME intakes is likely due to better apparent efficiency of energy utilisation by Mule ewes. However, no variation in CLW between Mule and Belclare X when offered WCWS suggests that Mule ewes did not adapt to WCWS as efficiently as they did on GS. This may be related to their ancestry, which is of hill origin where they have adapted to extensive grazing systems [49] compared to intensive micro-managed lowland systems such as winter feeding of whole crop cereal silages. The difference in CLW between Belclare X and Lleyn X ewes offered WCWS is an unexpected outcome as both Belclare X and Lleyn X ewes offered WCWS had no variation in ME intake per kg BW, ME % from forage, and apparent efficiency of energy utilisation, and warrants further investigation.

### 4.5. Colostrum Yield and Composition

Colostrum production potential is determined by energy and CP intake from feed, ewes’ mobilisation of body reserves [11,23,50] during late gestation as udder development undergoes 70% of all development in the final four weeks of gestation [51]. O’Doherty et al. [42] found that diets consisting of maize silage did not affect colostrum yield compared to diets consisting of grass silage, despite differences in dietary intake of CP, NDF, ADF, and starch, once both diets met the ewes energy and CP requirements. The findings of the current study are supported by O’Doherty et al. [42] showing that the nutrient supply, as opposed to the forage source, is the major driver of colostrum production.

McGovern et al. [11] reported increased total colostrum yield over the first 18 h postpartum as ME intake increased whereas Campion et al. [23] found no colostrum production response to increasing energy intake because ewes offered lowered energy levels responded by increasing body reserve mobilisation to compensate for nutrient intake deficits. Similarly, inadequate CP nutrition reduces colostrum yield [52] and is linked to reduced utilisation of starch and other nutrients [50], or excessive CP supply can lead to toxic rumen ammonia concentrations [46]. In the current study, CP supply was sufficient to meet 100% of ewe requirements [21].

Increased prolificacy is a key factor for increased farm profitability [20], however increased mortality can be a concurrent response to increased prolificacy [53] with this problem exacerbated if ewes are unable to produce sufficient colostrum to feed the increased number of lambs born [54]. The lower colostrum yields (total to 18 h postpartum and per kg BW) of the Mule ewes compared to Belclare X ewes concurs with the findings of Campion et al. [55] with consistent colostrum yields at 1 h postpartum in both studies. No significant differences in colostrum yield at 1 h postpartum were reported in the current study due to the large variation in colostrum yield at this time [23]. Mule ewes had reduced ME intake during late gestation and whilst this did not negatively impact BW, BCS, or CLW, the consequences of lower feed intake were observed in the reduced yield of colostrum compared to the other breed types. At 1 h, colostrum yield was only 63% compared to the yield of the other breed types, 31% of Mule ewes failed to produce 20 mL·kg^−1^ CLW and only 23% of Mule ewes reached the target of 50 mL·kg^−1^ CLW at 1 h postpartum. This indicates significant potential for challenge at farm level, especially in high challenge environments such as outdoor lambing in poor weather conditions. Lower ME intakes by Mule ewes with no difference in BW, BCS, or CLW subsequently resulted in reduced colostrum yield compared to the other breed types as energy intake is a major factor in colostrum production [11]. Therefore, lambs from Mule ewes received lower volumes of colostrum per kg birth weight than lambs from Belclare X ewes at 1 h postpartum, which is the crucial time for colostrum intake of the neonatal lamb [55] to consume sufficient energy, antibodies, and maintain homeothermy [56].

Ewe colostrum CP and TS concentration were unaltered over a range of diets and ME concentrations [11,23,52], which supports findings of the current study except at 10 h postpartum. At this time point, ewes offered WCWS displayed higher TS concentration than ewes offered GS as a result of lower colostrum yield changes between 1 and 10 h in agreement with Pattinson and Thomas [57] who stated a similar trend between meat breed types with lower colostrum yields over a 4 h period equating to increased TS concentration. Lower yield per hour can occur from high progesterone levels, which results in lower lactose concentration and subsequently lower colostrum yield [58]. Furthermore, offering WCWS to dairy cows did not illicit changes in milk composition [32].

Previous studies have found breed type effects on colostrum composition [55,57,59] as well as late gestation feeding. Pattinson and Thomas [57] reported that Lleyn X ewes had increased colostrum concentration of TS and fat per hour (measured between 12 and 16 h postpartum) compared to Cambridge X ewes with similar colostrum yield per hour. However, in the current study, with similar colostrum yields, Lleyn X ewes produced lower TS concentration at 10 h postpartum than other breed types. Crude protein concentration in colostrum was higher in Mule ewes at 1 and 10 h compared to Lleyn X ewes. This was expected as Mule ewes showed a tendency towards higher TS at 1 h and had significantly higher TS at 10 h compared to Lleyn X ewes (crude protein is the second main constituent in TS). Findings reported by Kessler et al. [59] concur with the current study that breed type affects CP as they reported differences between milk and meat breed types during the first 4h postpartum. While immunoglobulin was not measured in the current study, Campion et al. [55] reported reduced immunoglobulin G (IgG) yields in colostrum for Leicester X ewes at 1 h postpartum compared to other breed types. Immunoglobulin G is important for neonatal survival and reduced IgG in Leicester X ewes was a cause for concern [55] especially combined with lower colostrum yields by Mule ewes in the current study.

### 4.6. Lamb BW and Growth Rate

Lamb growth for the first eight weeks of life is closely correlated to nutrition that it receives from the ewe’s milk. McGovern et al. [44] and Campion et al. [23] reported strong links between increased energy intake prepartum, estimated milk production, and subsequent lamb growth for the first 14 weeks of life. Hutton et al. [60] reported that milk production and subsequent lamb performance was driven by diet type and nutrient intake postpartum when ewes were offered a single diet prepartum. This study was isoenergic during gestation, which explained the similarities reported in lamb BW and average daily gain throughout the lactation period between forage and breed types. These findings suggest that there were no negative effects from the alternative feed source (WCWS) with differing concentrations of CP, NDF, and starch during late gestation on subsequent lamb performance.

## 5. Conclusions

The findings of this study indicate that whole crop wheat silage can be offered to ewes as an alternative to grass silage during the final eight weeks of gestation but supplementation of concentrates may be required to ensure adequate protein in the ewes’ diet due to the low CP concentration in WCWS. Ewes allocated WCWS produced lambs of lower birthweight relative the GS fed animals, but all lamb birth weights were above standard industry targets. Additionally, Mule ewes had a higher apparent efficiency of energy utilisation compared to Belclare X and Lleyn X. However, colostrum yield, a crucial aspect of sheep production systems for maximising healthy and thriving lambs, was lower in Mule ewes compared to the other breed types. Breed type warrants further research to investigate whether Mule ewes are genetically predisposed to lower colostrum yields and lower feed intakes without impacting on maternal body condition score compared to other breed types.

## Figures and Tables

**Table 1:** Chemical composition of forages and concentrates offered to ewes during the final eight weeks of gestation.

Composition, g·kg^−1^ DM ^1^	Grass Silage	Whole Crop Wheat Silage	Concentrate
Dry matter, g·kg^−1^ FW	229.4	367.9	847.6
Crude protein	133.5	95.7	193.5
Neutral detergent fibre	430.6	355.5	150.5
Acid detergent fibre	259.7	202.0	76.4
Acid detergent lignin	26.7	22.9	5.3
Organic matter digestibility	712.3	677.7	865.9
Starch	-	229.0	273.4
Ash	71.4	48.0	79.4
Ether extract	32.0	30.6	28.3
pH, pH units	3.74	3.85	-
Gross energy, MJ·kg^−1^ DM	17.28	17.24	17.15
Metabolisable energy, MJ·kg^−1^ DM	10.93	10.53	12.79

^1^ DM = dry matter.

**Table 2:** The effect of forage type and breed type on ewe average daily dry matter (DM), metabolisable energy (ME), crude protein (CP), neutral detergent fibre (NDF) intake, and apparent efficiency of energy utilisation (least square means ± SEM).

Parameters	Forage Type ^1^				Breed Type				
	GS	WCWS	SEM	*p*-Value	Belclare X	Lleyn X	Mule	SEM	*p*-Value
Mean ^2^ daily DM intake, kg	1.22	1.25	0.030	>0.05	1.30 ^a^	1.26 ^a^	1.15 ^b^	0.034	<0.05
Mean DM intake per kg BW ^3^, g	14.98	15.32	0.409	>0.05	15.61 ^a^	15.69 ^a^	14.13 ^b^	0.985	<0.01
Mean daily ME intake, MJ	13.8	13.9	0.32	>0.05	14.5 ^a^	14.1 ^a^	12.9 ^b^	0.4	<0.01
Mean ME intake per kg BW, MJ	0.170	0.170	0.0041	>0.05	0.174 ^a^	0.178 ^a^	0.159 ^b^	0.0051	<0.01
Mean daily CP intake, g	175	144	3.4	<0.0001	166 ^a^	162 ^a^	151 ^b^	4.1	<0.05
Mean CP intake per kg BW, g	2.15	1.17	0.045	<0.0001	2.00 ^a^	2.02 ^a^	1.86 ^b^	0.055	<0.05
Mean daily NDF intake, g	468	398	12.2	<0.0001	455 ^a^	446 ^a^	400 ^b^	15.0	<0.01
Mean NDF intake per kg BW, g	5.75	4.87	0.163	<0.0001	5.47 ^a^	5.54 ^a^	4.92 ^b^	0.2	<0.01
Mean ME from forage ^4^, %	81.0	76.2	1.31	<0.001	79.7 ^a^	80.1 ^a^	76.1 ^b^	1.6	<0.05
Mean apparent efficiency of energy utilisation ^5^, %	106	115.3	3.29	<0.01	105.3 ^a^	105.3 ^a^	121.3 ^b^	4.03	<0.001

^1^ GS = grass silage; WCWS = whole crop wheat silage. ^2^ Mean = day 91 to day 147 of gestation ^3^ BW = body weight ^4^ ME from forage = percentage of ME intake from forage of total ME consumed by ewes ^5^ Mean apparent efficiency of energy utilisation = Ewe predicted required daily ME intake for actual CLW/Ewe actual daily ME intake ^a,b^ Within rows, means with differing superscripts differ significantly.

**Table 3:** The effect of forage type and breed type on ewe water intake during late gestation (least square means ± SEM).

Parameters	Forage Type ^1^				Breed Type				
	GS	WCWS	SEM	*p*-Value	Belclare X	Lleyn X	Mule	SEM	*p*-Value
Voluntary intake, L·d^−1^									
Day 113–115 of gestation	0.60 ^x^	2.83 ^x^	0.306	<0.0001	1.73 ^x^	2.06 ^x^	1.36 ^x^	0.375	>0.05
Day 126–128 of gestation	1.97 ^y^	4.60 ^y^	0.396	<0.0001	3.23 ^y^	3.80 ^y^	2.83 ^y^	0.485	>0.05
Day 141–143 of gestation	2.83 ^z^	3.98 ^z^	0.381	<0.05	3.26 ^y^	4.06 ^y^	2.89 ^y^	0.466	>0.05
SEM	0.209	0.211			0.260	0.250	0.261		
*p*-value	<0.0001	<0.01			<0.0001	<0.0001	<0.0001		
Mean	1.80	3.80	0.319	<0.0001	2.74 ^ab^	3.31 ^a^	2.36 ^b^	0.390	<0.05
In feed, L·d^−1^									
Day 113–115 of gestation	3.55 ^x^	1.82 ^x^	0.095	<0.0001	2.80 ^x^	2.80 ^x^	2.45 ^x^	0.116	>0.05
Day 126–128 of gestation	3.50 ^x^	1.56 ^y^	0.122	<0.0001	2.70 ^a,x^	2.77 ^a,x^	2.13 ^b,y^	0.150	<0.05
Day 141–143 of gestation	2.72 ^y^	1.28 ^z^	0.159	<0.0001	2.01 ^y^	2.02 ^y^	1.98 ^y^	0.195	>0.05
SEM	0.086	0.087			0.107	0.103	0.107		
*p*-value	<0.0001	<0.05			<0.0001	<0.0001	<0.05		
Mean	3.26	1.56	0.106	<0.0001	2.50 ^a^	2.53 ^a^	2.19 ^b^	0.130	<0.05
Water intake, L·d^−1^									
Day 113–115 of gestation	4.16 ^x^	4.64 ^x^	0.380	>0.05	4.53 ^x^	4.88 ^x^	3.80 ^x^	0.465	>0.05
Day 126–128 of gestation	5.46 ^y^	6.16 ^y^	0.381	>0.05	5.93 ^ab,y^	6.58 ^a,y^	4.92 ^b,y^	0.466	<0.05
Day 141–143 of gestation	5.56 ^y^	5.25 ^x^	0.380	>0.05	5.27 ^xy^	6.10 ^y^	4.86 ^y^	0.465	>0.05
SEM	0.227	0.229			0.282	0.272	0.284		
*p*-value	<0.0001	<0.001			<0.0001	<0.01	<0.05		
Mean	5.06	5.35	0.331	>0.05	5.24 ^ab^	5.85 ^a^	4.53 ^b^	0.405	<0.01
Water intake, per kg DM									
Day 113–115 of gestation	3.69	4.18	0.291	>0.05	3.73	4.30	3.78	0.356	>0.05
Day 126–128 of gestation	3.63	4.27	0.226	>0.05	3.75	4.29	3.81	0.277	>0.05
Day 141–143 of gestation	4.04	4.13	0.308	>0.05	3.64	4.47	4.15	0.377	>0.05
SEM	0.179	0.181			0.222	0.215	0.223		
*p*-value	>0.05	>0.05			>0.05	>0.05	>0.05		
Mean	3.79	4.20	0.234	>0.05	3.71	4.35	3.91	0.289	>0.05

^1^ GS = grass silage; WCWS = whole crop wheat silage. ^a,b^ Within rows, means with differing superscripts differ significantly ^x–z^ Within columns, means with differing superscripts differ significantly.

**Table 4:** The effects of forage type and breed type on ewe body weight (BW) and body condition score (BCS) and combined litter weight (CLW; least square means ± SEM).

Parameters	Forage Type ^1^				Breed Type				
	GS	WCWS	SEM	*p*-Value	Belclare X	Lleyn X	Mule	SEM	*p*-Value
BW, kg									
Pre-experiment	81.43 ^w^	81.94 ^wx^	1.873	>0.05	83.53 ^wx^	79.56 ^wx^	81.96 ^wx^	2.294	>0.05
Mid-experiment	84.04 ^x^	83.40 ^w^	1.872	>0.05	85.50 ^w^	81.69 ^w^	84.01 ^w^	2.293	>0.05
Parturition	79.02 ^y^	79.87 ^xy^	1.873	>0.05	81.59 ^x^	77.89 ^xy^	78.86 ^xy^	2.294	>0.05
Day 21 postpartum	78.50 ^y^	78.24 ^y^	1.890	>0.05	80.94 ^x^	75.92 ^y^	78.24 ^y^	2.315	>0.05
Day 42 postpartum	74.56 ^z^	73.85 ^z^	1.891	>0.05	76.90 ^y^	70.55 ^z^	75.16 ^z^	2.315	>0.05
Weaning (Day 98 postpartum)	73.19 ^z^	72.39 ^z^	1.904	>0.05	74.66 ^y^	69.53 ^z^	74.17 ^z^	2.330	>0.05
SEM	0.722	0.751			0.897	0.879	0.930		
*p*-value	<0.05	<0.001			<0.001	<0.05	<0.05		
Pre-experiment to parturition, % change	−2.84	−2.55	1.064	>0.05	−2.31	−2.06	−3.71	1.302	>0.05
Pre-experiment to weaning, % change	−9.86	−11.48	1.535	>0.05	−10.54	−12.32	−9.15	1.872	>0.05
BCS, BCS units									
Pre-experiment	3.20 ^x^	3.28 ^v^	0.098	>0.05	3.19 ^x^	3.20 ^x^	3.36 ^x^	0.120	>0.05
Mid-experiment	3.00 ^xy^	3.20 ^vw^	0.097	>0.05	3.05 ^xy^	3.07 ^xy^	3.18 ^xy^	0.119	>0.05
Parturition	2.88 ^yz^	2.98 ^y^	0.097	>0.05	2.84 ^yz^	2.98 ^xyz^	2.97 ^yz^	0.119	>0.05
Day 21 postpartum	2.82 ^yz^	2.65 ^x^	0.099	>0.05	2.61 ^z^	2.82 ^yz^	2.77 ^z^	0.122	>0.05
Day 42 postpartum	2.70 ^z^	2.72 ^xz^	0.100	>0.05	2.71 ^z^	2.74 ^z^	2.69 ^z^	0.122	>0.05
Weaning (Day 98 postpartum)	2.88 ^yz^	2.95 ^wyz^	0.101	>0.05	2.86 ^yz^	2.99 ^xyz^	2.90 ^yz^	0.124	>0.05
SEM	0.733	0.076			0.091	0.089	0.094		
*p*-value	<0.01	< 0.05			<0.05	<0.05	<0.01		
Pre-experiment to parturition, % change	−9.38	−8.22	3.457	>0.05	−9.70	−5.54	−11.16	4.231	>0.05
Pre-experiment to weaning, % change	−8.38	−8.46	3.425	>0.05	−8.15	−4.43	−12.67	4.177	>0.05
CLW	10.47	9.83	0.279	<0.05	10.22	9.86	10.36	0.342	>0.05
kg of CLW per ewe BW ^2^, %	13.32	12.48	0.399	<0.05	12.63	12.87	13.2	0.488	>0.05

^1^ GS = grass silage; WCWS = whole crop wheat silage ^2^ BW 24 h postpartum ^v–z^ Within columns, means with differing superscripts differ significantly.

**Table 5:** The effect of forage type and breed type on colostrum yield and composition (least square means ± SEM).

Parameters	Forage Type ^1^				Breed Type				
	GS	WCWS	SEM	*p*-Value	Belclare X	Lleyn X	Mule	SEM	*p*-Value
Colostrum yield, mL									
1 h postpartum	541 ^x^	543	73.6	>0.05	635	602	389 ^x^	90	>0.05
10 h postpartum	692 ^y^	628	54.5	>0.05	708	623	650 ^y^	66.6	>0.05
18 h postpartum	683 ^xy^	618	51.2	>0.05	672	610	669 ^y^	62.7	>0.05
SEM	44.3	45.7			71.2	54.2	56.2		
*p*-value	<0.05	>0.05			>0.05	>0.05	<0.01		
Total	1953	1763	137.9	>0.05	2011 ^a^	1895 ^ab^	1668 ^b^	168.9	<0.05
Colostrum composition, mL·L^−1^									
TS ^2^									
1 h postpartum	382 ^x^	411 ^x^	17.0	>0.05	374 ^x^	376 ^x^	440 ^x^	20.9	>0.05
10 h postpartum	297 ^y^	340 ^y^	14.4	<0.05	310 ^ab,y^	292 ^a,y^	353 ^b,y^	17.6	<0.05
18 h postpartum	233 ^z^	258 ^z^	11.0	>0.05	235 ^z^	230 ^z^	272 ^z^	13.4	>0.05
SEM	8.8	9.2			10.9	10.7	11.6		
*p*-value	<0.0001	<0.0001			<0.0001	<0.0001	< 0.0001		
CP ^3^									
1 h postpartum	197 ^x^	212 ^x^	7.5	>0.05	200 ^ab,x^	192 ^a,x^	221 ^b,x^	9.1	<0.05
10 h postpartum	127 ^y^	146 ^y^	7.3	>0.05	130 ^ab,y^	123 ^a,y^	156 ^b,y^	9.0	<0.01
18 h postpartum	78 ^z^	89 ^z^	7.3	>0.05	80 ^z^	77 ^z^	93 ^z^	9.0	>0.05
SEM	4.8	5.1			6.0	5.9	6.4		
*p*-value	<0.0001	<0.0001			<0.0001	<0.0001	<0.0001		
Total colostrum produced per kg of ewe BW, mL	24.7	22.4	1.87	>0.05	25.1	24.4	21.2	2.29	>0.05
Total colostrum produced per kg of CLW ^4^, mL	187	180	13.2	>0.05	198 ^a^	192 ^ab^	160 ^b^	16.2	<0.05
Colostrum per hour between 1–10 h, mL	77.8	69.6	6.02	>0.05	78.6	71.5	71.0	7.36	>0.05
Colostrum per hour between 10–18 h, mL	85.0	77.9	6.51	>0.05	85.1	76.3	83.0	7.99	>0.05

^1^ GS = grass silage; WCWS = whole crop wheat silage ^2^ TS = total solid ^3^ CP = crude protein ^4^ CLW = combined litter weight ^a,b^ Within rows, means with differing superscripts differ significantly ^x–z^ Within columns, means with differing superscripts differ significantly.

**Table 6:** The effect of forage type and breed type on lamb colostrum intake per kg of birth weight ^1^ (least square means ± SEM).

Parameters	Forage Type ^2^				Breed Type				
	GS	WCWS	SEM	*p*-Value	Belclare X	Lleyn X	Mule	SEM	*p*-Value
1 h postpartum, mL	39.5 ^x^	35.2 ^x^	2.47	>0.05	43.5 ^a^	41.3 ^ab^	27.2 ^b,x^	3.02	<0.001
10 h postpartum, mL	46.9 ^y^	44.5 ^y^	1.39	>0.05	47.3	46.3	43.5 ^y^	1.70	>0.05
18 h postpartum, mL	46.6 ^y^	45.8 ^y^	1.06	>0.05	47.3	46.7	44.6 ^y^	1.30	>0.05
SEM	1.40	1.44			1.71	1.73	1.77		
*p*-value	<0.001	<0.0001			>0.05	>0.05	<0.0001		
Total ^3^	133.1	125.5	3.76	<0.05	138.2 ^a^	134.3 ^a^	115.5 ^b^	4.60	<0.01

^1^ only includes colostrum received from the birth dam of each lamb ^2^ GS = grass silage; WCWS = whole crop wheat silage ^3^ Combined total of colostrum received over the 18 h period ^a,b^ Within rows, means with differing superscripts differ significantly ^x,y^ within columns, means with differing superscripts differ significantly.

**Table 7:** The effect of forage type and breed type on lamb body weight (BW) and average daily gain (ADG; least square means ± SEM).

Parameters	Forage Type ^1^				Breed Type				
	GS	WCWS	SEM	*p*-Value	Belclare X	Lleyn X	Mule	SEM	*p*-Value
Lamb weights, kg									
Birth weight	5.21	5.01	0.119	>0.05	5.11	4.95	5.26	0.146	>0.05
21 days of age	12.07	11.78	0.280	>0.05	12.00	11.63	12.14	0.342	>0.05
42 days of age	16.85	16.48	0.348	>0.05	16.93	16.22	16.86	0.425	>0.05
Weaning weight (98 days of age)	27.64	26.78	0.526	>0.05	26.98	26.93	27.70	0.642	>0.05
Overall ADG ^2^, g	242.4	236.7	5.47	>0.05	239.8	236.2	242.6	6.95	>0.05

^1^ GS = grass silage; WCWS = whole crop wheat silage ^2^ ADG is calculated as a regression of BW on time.
